# STEPWISE CARDIAC REHABILITATION ADJUSTMENT AFTER EXERCISE-INDUCED IMPLANTABLE CARDIOVERTER DEFIBRILLATOR SHOCK: A CASE REPORT

**DOI:** 10.2340/jrm-cc.v8.42483

**Published:** 2025-04-16

**Authors:** Hidetoshi YANAGI, Harumi KONISHI, Saori YAMADA, Kazuya YAMAMOTO, Fumiyuki OTSUKA

**Affiliations:** 1 Department of Cardiovascular Rehabilitation, National Cerebral and Cardiovascular Center, Suita, Japan; 2 Department of Nursing, National Cerebral and Cardiovascular Center, Suita, Japan; 3 Department of Cardiovascular Medicine, National Cerebral and Cardiovascular Center, Suita, Japan

**Keywords:** exercise, implantable cardioverter defibrillator, ventricular arrhythmia

## Abstract

**Objective:**

To report an in-hospital cardiac rehabilitation strategy after exercise-induced implantable cardioverter defibrillator shock.

**Case report:**

A 72-year-old man with heart failure, peripheral artery disease, a history of percutaneous coronary intervention and coronary artery bypass surgery, exercise-induced ventricular fibrillation, and an implantable cardioverter defibrillator was hospitalised after experiencing recurrent ventricular fibrillation while walking, which triggered implantable cardioverter defibrillator shock. While hospitalised, his medication regimen was adjusted. After passing the 200-m walking test, he started in-hospital cardiac rehabilitation. During cardiopulmonary exercise testing, he experienced non-sustained ventricular tachycardia. Percutaneous coronary intervention was performed to relieve ischaemia; however, ventricular tachycardia recurred during walking, causing another implantable cardioverter defibrillator shock. After further medication adjustments and setting heart rate limits, he gradually resumed cycling and low-intensity resistance exercises, followed by walking, and was subsequently discharged without ventricular tachycardia recurrence.

**Discussion:**

Peripheral artery disease-associated pain and increased heart rate may have contributed to ventricular tachycardia. A stepwise exercise programme involving heart rate monitoring and medication therapy adjustments enabled safe exercise resumption after implantable cardioverter defibrillator shock in a patient with multiple comorbidities.

**Conclusion:**

This case emphasises the importance of personalised exercise strategies that consider both arrhythmic risk and comorbidities for patients at high risk of exercise-induced arrhythmias.

## LAY ABSTRACT

A 72-year-old man with heart failure, chronic kidney disease, and peripheral artery disease experienced exercise-induced shocks from his implantable cardioverter defibrillator even while participating in a gradual rehabilitation programme. He was admitted to our facility after experiencing recurrent life-threatening irregular heart rhythms diagnosed as ventricular fibrillation while walking, which caused shocks from his implantable defibrillator. He initially participated in a cardiac rehabilitation programme but again experienced life-threatening irregular heart rhythms. After participating in a stepwise exercise programme in which his heart rate was monitored and his medications were adjusted gradually, the patient could safely resume his exercises. This case provides insights into the rehabilitation process after experiencing such shocks and presents new considerations for cardiac rehabilitation.

Currently, millions of patients worldwide have implantable cardioverter defibrillators (ICDs), and the number of ICD implantations continues to increase annually (1, 2). Consequently, many participants involved in comprehensive cardiac rehabilitation programmes now have ICDs (3). Meta-analyses have demonstrated that comprehensive these programmes can improve exercise capacity in patients with ICDs without causing adverse events (4, 5). Reports of ICD interventions during exercise therapy have included 1 case of anti-tachycardia pacing activation during home-based exercise therapy and 6 cases of ICD shocks occurring during supervised exercise therapy (6, 7). Furthermore, our recent retrospective study involving 524 patients with cardiac implantable electronic devices indicated that the incidence of ICD shocks during exercise therapy was 1 event per 5977 patient-hours (8). Despite this, limited guidance is available on managing ICD shocks that occur during exercise therapy and on safely resuming rehabilitation following such incidents. Cardiac rehabilitation not only improves exercise capacity but also provides an opportunity for psychological adaptation, cardiovascular risk management, and device function assessment in patients with ICDs (3). ICD recipients often fear shocks, which can lead to self-imposed restrictions on physical activity and reduced participation in rehabilitation (3). Given these unique challenges, further research is needed to establish safe and effective rehabilitation strategies for ICD patients.

We present the case of a patient with an ICD who experienced an ICD shock during a comprehensive cardiac rehabilitation programme.

## CASE DESCRIPTION

The patient was a 72-year-old man who had been diagnosed with triple vessel disease at 69 years of age at another hospital, 3 years before his admission to our facility. He was administered oral medication but discontinued hospital visits at his discretion. He was diagnosed with critical limb ischaemia caused by peripheral artery disease (PAD) at approximately the same time as this initial hospitalisation, leading to the amputation of his right fifth toe. The patient had no history of alcohol consumption, had smoked until the age of 68 years, and had no family history of sudden cardiac death.

At the age of 70 years, 2 years prior to hospitalisation at our facility, the patient underwent percutaneous coronary intervention (PCI) on segment 11 and segments 1–2 of the coronary arteries and segments 3–4 of the posterior descending coronary arteries, as well as endovascular therapy on the left below-the-knee and anterior tibial arteries. After these procedures, he discontinued hospital visits again.

At the age of 71 years, 1 year prior to hospitalisation at our facility, the patient had an episode of syncope caused by ventricular tachycardia (VT); he was admitted via the emergency department to another hospital and then transferred to our hospital, where he received an ICD (Evera MRI XT DR DDMB2D4; Medtronic Inc., Minneapolis, MN, USA). One month thereafter, the patient developed low cardiac-output syndrome triggered by an infection, and the VT recurred. He underwent emergency coronary artery bypass surgery (Ao-SVG-free LITA-LAD, Ao-SVG-PD-PL-PL) and aortic valve replacement with percutaneous cardiopulmonary support and intra-aortic balloon pumping. After the procedures, the patient was transferred to a rehabilitation hospital. During this time, stenosis of the left common femoral artery was also observed.

**Fig. 1:**
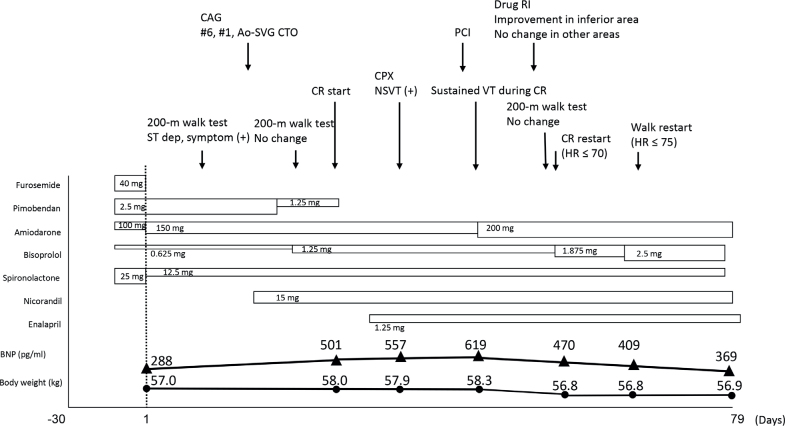
Case history. AoSVG: aorta–saphenous vein graft; BNP: B-type natriuretic peptide; CAG: coronary angiography; CPX: cardiopulmonary exercise test; CR: cardiac rehabilitation; CTO: chronic total occlusion; HR: heart rate; NSVT: non-sustained ventricular tachycardia; PCI: percutaneous coronary intervention; RI: radio isotope inspection; VT: ventricular tachycardia.

At the age of 72 years, 1 year after the previous hospitalisation, the patient experienced ventricular fibrillation (VF) while walking, and the ICD responded appropriately. The following month, VF recurred, resulting in emergency hospitalisation at our facility. After admission, his amiodarone dose was increased from 100 to 150 mg (Fig. 1). However, ST-T depression and chest symptoms appeared during the 200-m walk test. Coronary angiography revealed chronic total occlusion of segments 6 and 1, as well as the ostium of the aorta–saphenous vein graft; however, due to the presence of chronic kidney disease, drug therapy was initially selected. After administering 15 mg nicorandil, the 200-m walk test was repeated 7 days later, and no ST-T changes or chest symptoms were observed (Fig. 1). Therefore, on day 28 of hospitalisation, cardiac rehabilitation was initiated in the recovery phase to evaluate ventricular arrhythmias during exercise, assess exertional ischaemia for determining the need for PCI, and improve exercise tolerance. The patient’s primary concerns were understanding safe levels of physical activity and learning strategies to prevent the recurrence of ICD shocks. A submaximal treadmill exercise test revealed no ischaemic symptoms or arrhythmias.

At the start of cardiac rehabilitation, the patient had a Self-rating Depression Scale (SDS) score (9) of 58.75, indicating a moderate level of depressive symptoms. In addition, the Japanese version of the Medical Outcomes Study 36-item Short Form Health Survey questionnaire (SF-36) (10) physical component summary (PCS) score was 36.3, reflecting impaired physical health-related quality of life, while the mental component summary (MCS) score was 60.1, suggesting relatively preserved mental health.

At this time, the patient’s body mass index was 20.7 kg/m^2^. His left ventricle ejection fraction was 20%, left ventricular end-diastolic diameter was 65 mm, and left ventricular end-systolic diameter was 50 mm. The ICD settings were as follows: mode, AAI-DDD; lower rate, 60 bpm; upper rate, 105 bpm; VT detection zone, 115 bpm; and VF detection zone, 200 bpm.

The cardiac rehabilitation protocol at our hospital has been reported previously (8). In brief, track walking, cycling (initially with a low load), and low-intensity resistance training were conducted while perceived exertion was monitored using the Borg scale (11) until the cardiopulmonary exercise test was conducted. Patients also received self-care and behavioural guidance as part of the rehabilitation programme, including lifestyle education for ischaemic heart disease and heart failure (HF), provided by nurses. Heart rate (HR) monitoring during walking was performed using a “heart-walking pacemaker” (Asuka Electric Co., Ltd., Osaka, Japan), which allows patients to check their HRs on a screen while walking. Additionally, cycling was performed on a Well Bike BE360 ergometer (Fukuda Denshi, Tokyo, Japan), controlled by an ML3600 (Fukuda Denshi), with HR monitoring and load adjustment. A physical therapist directly supervised both walking and cycling sessions to ensure safe exercise intensity, prevent ICD shock risk, and adjust training loads accordingly. Additionally, a nurse and a physician were present on the same floor, ensuring immediate medical support if needed. In this patient, as presented in Table I, cycling intensity was increased in both work rate (wattage) and duration. Walking time varied from day to day due to PAD.

**Table I:** Exercise therapy program: detailed progression and parameters

Variable	Parameter value																								
Day	29	30	31	32	35	36	38	39	42	43	46	59	60	63	64	65	66	67	71	72	73	74	77	78	79
*Walking*																									
Walking distance, m	285	304	334	410	357	517	642	406	546	607	463								271	243	357	425	474	486	425
Time, min	5	5	5	7	7	9	10	6	8	10									5	7	7	8	8	8	8
Max HR, bpm	78	70	75	95	71	71	88	87	105	94									65	71	71	77	73	75	74
Borg scale (dyspnoea)	11	13	11	13	11	12	11	11	11	13									12	12	12	12	13	13	12
Borg scale (leg fatigue)	13	13	12	13	15	13	13	15	15	13									12	12	12	13	13	13	13
*Cycling (1)*																									
Work rate, Watts	10	10	10	10	10	10	20	20–25	20–25	20–25		10	10	10	10	15	20	20	20	25	25	25	25	25–30	25–30
Time, min	5	10	15	15	15	20	20	15	20	20		10	15	15	20	20	20	20	20	20	20	20	20	20	20
Max HR, bpm	66	86	66	73	61	61	70	70	70	71		60	66	62	61	60	66	63	60	69	65	71	67	74	68
Borg scale (dyspnoea)	11	11	11	12	11	12	12	12	12	12		11	12	12	12	11	12	11	12	12	12	12	12	12	12
Borg scale (leg fatigue)	11	12	11	12	11	12	12	13	11	12		11	12	12	12	11	12	11	12	13	12	12	12	12	12
*Cycling (2)*																									
Work rate, Watts												10	10	10	10	15	20	25							
Time, min												10	15	15	20	20	20	20							
Max HR, bpm												61	63	60	62	60	67	68							
Borg scale (dyspnoea)												11	12	12	12	11	12	13							
Borg scale (leg fatigue)												11	12	12	12	11	12	13							
*Resistance training*																									
Max HR, bpm	65		62	70	60	61	66		68			60		60		60		60	66			64	62		60
Borg scale (dyspnoea)	12		11	11	11	11	12		12			12		11		11		13	12			12	13		12
Borg scale (leg fatigue)	12		11	13	11	13	12		13			13		11		11		13	12			12	13		12

HR: heart rate; bmp: beats per minute.

**Fig. 2:**
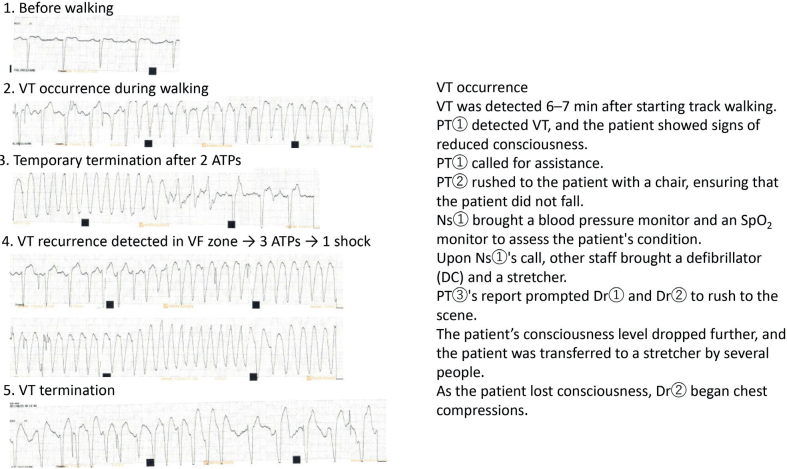
Advanced cardiac life support during cardiac rehabilitation. ATP: anti-tachycardia pacing; DC: direct current; Dr: doctor; Ns: nurse; PT: physical therapist; SpO_2_: peripheral oxygen saturation; VF: ventricular fibrillation; VT: ventricular tachycardia.

On day 37 of hospitalisation, a submaximal cardiopulmonary exercise test revealed non-sustained VT, and since the VT detection zone (> 115 bpm) had been reached, the test was discontinued. Revascularisation was planned, and PCI was performed on day 44 of hospitalisation. Shortly before PCI, the patient’s HR during walking ranged from 80 to 100 bpm, and the Borg scale score was high, at 13–15. During rehabilitation, on day 46, exercise-induced VT (HR, 95 bpm) occurred during walking. The team successfully performed appropriate secondary life-saving measures (Fig. 2).

After VT onset, the amiodarone dose was increased from 150 to 200 mg. Thereafter, no signs of HF exacerbation, involving body weight, B-type natriuretic peptide levels, or echocardiographic findings, were observed. Additionally, no new ischaemia was observed in the drug radioisotope inspection test.

Following the successful completion of the 200-m walk test, cardiac rehabilitation in the recovery phase was resumed on day 59 (13 days after VT onset). Initially, rehabilitation focused on improving exercise tolerance; however, after the onset of exercise-induced VT, the primary goal shifted to evaluating ventricular arrhythmias during exercise to ensure safe physical activity before discharge. To achieve this, exercise was resumed with an HR limit of ≤ 70 bpm, which was carefully monitored to prevent arrhythmic events. Additionally, the bisoprolol dose was increased from 1.25 to 1.875 mg, with a HR limit of ≤ 70 bpm observed during the 200-m walk test (Table I).

Only cycling and low-intensity resistance exercises were performed, and HR was maintained at ≤ 70 bpm, with intensity maintained at a Borg scale score of 11–12. A stationary bicycle was selected as the first option for exercise, as before the VT onset, neither Borg scale scores nor HR showed excessive increases during this exercise, and the bicycle facilitated workload quantification. On day 71 of hospitalisation, walking exercises were resumed, and the HR limit was increased to 75 bpm. By performing walking exercises while paying attention to the HR limit, the patient was able to perform exercises at a Borg scale score of 12–13, without excessive HR elevation. No non-sustained VT occurred after resumption of cardiac rehabilitation, and the patient was discharged on day 80 of hospitalisation.

## DISCUSSION

We have described the case of a patient with ischaemic HF, complicated by chronic kidney disease and PAD, who had previously undergone PCI and coronary artery bypass surgery. The patient had a history of exercise-induced VF. During hospitalisation, the medication was adjusted, and the patient started a phased cardiac rehabilitation programme. However, VT recurred during walking, leading to ICD shock.

Several factors may have triggered VT onset in this patient. First, the rate of recurrence of ventricular arrhythmias in patients with HF has been reported to be 10–20% (12). HF can trigger arrhythmias, and in turn, ventricular arrhythmias can exacerbate HF by accelerating its progression (13). In this case, by monitoring the patient’s body weight, B-type natriuretic peptide level, and echocardiographic findings throughout hospitalisation, we determined that HF exacerbation was unlikely at the time of VT onset. Second, while the possibility of the effects of residual ischaemia cannot be ruled out, no new ischaemic events were observed, and further revascularisation procedures were deemed difficult. Third, ICD shock rarely occurs during exercise (7, 8). However, this patient had a history of VT/VF and severe arrhythmias during exercise stress testing, making him a high-risk candidate for ICD shock (14). Finally, this patient experienced pain during walking due to PAD, which likely triggered an increase in HR and subsequently induced VT. At that time, high-intensity walking exercise was recommended for patients with PAD, which may have prompted increased exercise intensity during walking.

ICD shocks during exercise are rare, and information on strategies for resuming rehabilitation after such incidents is scarce. However, a previous report recounted the cases of 6 individuals who had experienced ICD shocks, with 4 of them continuing rehabilitation (7). After undergoing ICD implantation, modifications to the treatment strategy, including device settings, pharmacotherapy, and exercise, should be considered (15). Simultaneously, prompt resumption of exercise is recommended to avoid psychological barriers to future physical activities (3).

Following VT onset in our patient, the doses of bisoprolol and amiodarone were increased, and recovery phase rehabilitation was restarted with cycling. An upper limit for HR was set to facilitate easier quantification of exercise intensity, and the rehabilitation progressed gradually, in stages. By the time of discharge, the patient was able to perform walking exercises with a Borg scale score of 12–13 without excessive HR elevation, suggesting a stable adaptation to exercise within monitored conditions.

A recent meta-analysis suggested that low- to moderate-intensity exercise improves maximal walking distance more than does high-intensity exercise in patients with PAD (16). Owing to the patient’s history of exercise-induced arrhythmias, moderate- to high-intensity exercise was avoided, and HR limits were carefully monitored, leading to a safe discharge without VT recurrence (3). Psychological factors play a crucial role in the rehabilitation of ICD recipients, as fear of exercise-induced ICD shock is a well-documented barrier to rehabilitation participation. In this case, the patient exhibited moderate depressive symptoms (SDS: 58.75) and impaired physical quality of life (SF-36 PCS: 36.3) at the start of rehabilitation, while mental health (SF-36 MCS: 60.1) was relatively maintained. These psychological challenges further highlight the importance of tailored rehabilitation strategies to ensure long-term adherence to exercise therapy.

For patients with a history of VT/VF, gradual rehabilitation and careful management of exercise intensity (e.g. HR limits and Borg scale score) are essential to reduce the risk of ICD shocks. In high-risk patients with overlapping conditions, a comprehensive evaluation of the risks, including triggering arrhythmias, and the benefits of rehabilitation, such as improving exercise tolerance, is critical for developing a safe and effective treatment strategy.

In conclusion, this case report described a rare occurrence of ICD activation during exercise therapy in a patient with ischaemic HF, chronic kidney disease, and PAD. Despite a carefully managed rehabilitation programme, the patient experienced VT, necessitating adjustments to the treatment strategy. Gradual resumption of cardiac rehabilitation with HR monitoring and pharmacological adjustments led to a safe discharge without VT recurrence. Thus, this case emphasises the importance of tailored rehabilitation protocols and close monitoring of high-risk patients.
